# Opto-thermally Excited Fabry-Perot Resonance Frequency Behaviors of Clamped Circular Graphene Membrane

**DOI:** 10.3390/nano9040563

**Published:** 2019-04-07

**Authors:** Fu-Tao Shi, Shang-Chun Fan, Cheng Li, Zi-Ang Li

**Affiliations:** 1 School of Instrumentation and Optoelectronic Engineering, Beihang University, Beijing 100191, China; liziang@buaa.edu.cn; 2 Shenzhen Institute of Beihang University, Shenzhen 518063, China

**Keywords:** graphene membrane, Fabry-Perot resonator, opto-thermal actuation, resonance frequency simulation

## Abstract

An opto-thermally excited optical fiber Fabry-Perot (F-P) resonant probe with suspended clamped circular graphene diaphragm is presented in this paper. Then, the dependence of resonance frequency behaviors of graphene diaphragm upon opto-mechanical factors including membrane properties, laser excitation parameters and film boundary conditions are investigated via COMSOL Multiphysics simulation. The results show that the radius and thickness of membrane will linearly affect the optical fiber light-induced temperature distribution, thus resulting in rapidly decreasing resonance frequency changes with the radius-to-thickness ratio. Moreover, the prestress can be regulated in the range of 10^8^ Pa to 10^9^ Pa by altering the environmental temperature with a scale factor of 14.2 MPa/K. It is important to note that the availability of F-P resonant probe with a defective clamped circular graphene membrane can be improved notably by fabricating the defected circular membrane to a double-end clamped beam, which gives a broader perspective to characterize the resonance performance of opto-thermally excited F-P resonators.

## 1. Introduction

Graphene is a single atomic plane of graphite, which is the thinnest and strongest two-dimensional material in the universe known up to now [1,2,3,4]. Due to its extraordinary electronic, mechanical, thermal, optical and optoelectronic properties, research about the new material have expanded explosively in the development of microelectromechanical system (MEMS) field, such as high frequency transistors, transparent conductors and mechanical resonators [5,6,7,8,9]. In fact, graphene shows extremely high Young modulus (1.1 TPa) and it can be stretched by 20% [10], which makes it appropriate for a sensitive element to develop high performance resonant sensors. Note that Bunch et al. started the study on the natural frequency and the quality factor (*Q*) of suspended single-layer graphene sheet over trenches, which indicated the feasibility of resonant graphene sensors [11]. Then, Chen et al. investigated the mechanical vibration of a monolayer nano-mechanical resonator by electrical detecting [12]. In addition, Oshidari et al. reported a graphene resonator by attaching SU-8 proof mass onto graphene, and studied the effect of temperature on the resonance frequency and quality factor variations of the resonator [13]. Recently, Kown et al. further investigated the mechanical response of graphene harmonic oscillator under nano-indentation by molecular dynamics method, which indicates that the resonance behaviors of graphene resonator can be tuned by nano-pressure and initial displacement [14,15]. Although currently, graphene mechanical resonators mainly adopt electrical excitation to actuate the sensitive elements [11,12,13,14,15,16], this type of excitation mode is generally limited to high measurement accuracy and better repeatability.

In contrast, opto-thermal excitation is a more efficient actuation mechanism for suspended resonant structures utilizing thermomechanical coupled [17,18], because it is a non-contact technique that can be implemented at lower micron and nanometer scale without sophisticated material integration. For example, recently Ramos et al. and Bonaldi et al. have, respectively, developed silicon nitride membrane resonators to probe optomechanical characterization of nanoscale microdrum resonators, which further indicates the advantage of optomechanical detection and actuation [19,20]. Furthermore, the availability of graphene acting as an excellent material with extraordinary opto-electronic properties and extremely high thermal conductivity [21,22,23,24] can provide the possibility of exploring the ability to absorb the light for optoelectronics application, along with the benefit of the ease of being optically tunable in intensity and frequency [25,26]. For example, in 2008, Ghosh et al. measured the thermal conductivity of graphene suspended across on Si/SiO_2_ wafer actuated by a laser with a wavelength of 488 nm, therefore suggesting graphene’s application as thermal management material in nanoelectromechanical system (NEMS) [27]. Additionally, Balandin et al. reported the measurement of thermal conductivity of a suspended single-layer graphene by optical excitation, which showed that the graphene could be used as an excellent element in opto-thermal excitation system [28]. Additionally, Xu et al. studied the thermal conduction in suspended monolayer graphene as a function of the temperature and length, which further supported the fundamental understanding of thermal transport in two-dimensional materials [29]. Although a large portion of the research has utilized optical technique to probe the thermal conductivity and thermal conduction of suspended graphene, few studies have focused on the investigation of resonance properties of graphene, especially the exploration of graphene resonators actuated by optical fiber F-P actuation and detection method.

In reality, in 2013, Lee et al. observed the intrinsic thermomechanical modes of the MoS_2_ resonators at room temperature and then performed the thermomechanical resonance measurement, which offered a support for development of opto-thermally excited graphene resonator [30]. Recently, Li et al. explored the opto-mechanical behaviors of MoS_2_ nanomembrane resonator in air by using optical F-P interferometric excitation and detection method [31]. They also fabricated a miniature micro-air-gap-based optical fiber F-P graphene resonator for experimentally characterizing the vibration behaviors of pressure-induced graphene membrane at room temperature [32]. Then, Dolleman demonstrated the parametric resonance responses of suspended single-layer graphene membrane via an opto-thermal driver modulating the intrinsic spring constant [33]. Unfortunately, these aforementioned studies have not yet given a deep insight into the dependence of optical-mechanical parameters on resonance characteristics of opto-thermally actuated graphene resonators. 

Therefore, in order to advance opto-thermal applications in graphene F-P resonators, in combination with the fabricated resonant probe with clamped circular graphene membrane, the relations between the membrane structural properties, optical excitation parameter and membrane boundary condition the and resonance frequency response were investigated by COMSOL Multiphysics simulation in this paper. The simulation results illustrated that the prestressed F-P resonator exhibited an excellent linearity in a prestress range of 10^8^ Pa to 10^9^ Pa, wherein the prestress was available to be tuned by changing the temperature ranging from 293.25 K (room temperature) to 393.15 K. Compared to those with asymmetrical defects at the edge of membrane, the F-P resonator probes under bilateral symmetry boundary conditions obtained better vibration modes when an unstable clamped boundary was turned into a stable double-end fixed one, thereby significantly enhancing the performance of F-P resonators.

## 2. The Model Adapted to Opto-Thermal F-P Resonance Measurement

**Figure 1:**
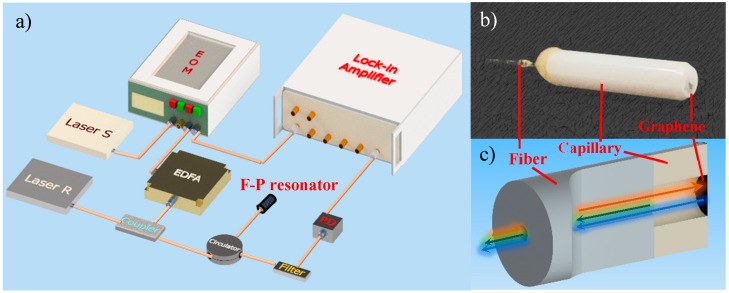
(**a**) Schematic illustration of opto-thermal actuation setup for resonance measurement; (**b**) picture and (**c**) physical diagram of the developed F-P resonant probe.

Figure 1a illustrates the schematic diagram of the optical excitation and detection method utilizing an F-P resonant probe. The initial cavity length between the fiber end and ferrule endface for the developed F-P resonant probe was confirmed as ~40 μm by an optical spectrum analyzer (AQ6370C, Yokogawa Electric Corporation, Tokyo, Japan) to generate a stable F-P interference pattern. After that, there is no need to sweep in the graphene/fiber endface distance. A distributed feedback (DFB) laser S with a wavelength of 1550.12 nm (10-dB output power) was modulated by an electro-optic modulator (EOM), which was used to impose a thermal excitation upon the circular graphene membrane. The laser S emitted a cosine wave via a lock-in amplifier (HL2FI). Then, an erbium doped fiber amplifier (EDFA) was added to compensate the amplitude attenuation of laser S modulated by EOM. Another DFB laser R with a wavelength of 1551.72 nm (10-dB output power) was employed to detect the vibration of membrane. The light signal generated by the laser R was optically coupled with that by the laser S that was amplified by the EDFA through a 2 × 1 coupler. The coupled signals were delivered to the F-P cavity of resonant probe through a circulator and then irradiated to the surface of graphene membrane suspended on a capillary endface. Then, the detection signal was reserved, while the excitation light was filtered by an optical filter. The filtered detection signal was sent to a lock-in amplifier through a photoelectric detector (PD, Beijing Conquer Optics Science & Technology Co., Ltd, Beijing, China) with a bandwidth of 200 MHz and a conversion gain of 1.4 × 10^4^ V/W for observing and processing of useful F-P resonance signals. 

With the use of the presented F-P resonant probe, the obtained natural frequency of 50-μm diameter circular graphene membrane with a thickness of 3.35 nm was confirmed as 1.7 MHz, which is much higher than the resonance frequency of 17 kHz in previously reported F-P resonator with a 125-μm diameter graphene film [32]. Unfortunately, there is no apparent enhancement in quality factor (*Q* = 4.81) compared to the measured *Q* value of 3.37 in our previous work [32]. Among those opto-mechanical factors influencing the resonance characteristics of the F-P probe, the structural property, film prestress, laser excitation parameter and boundary condition of elastic sensitive element are essential to understand and optimize the resonant behaviors of the photo-thermally actuated graphene membrane.

**Figure 2:**
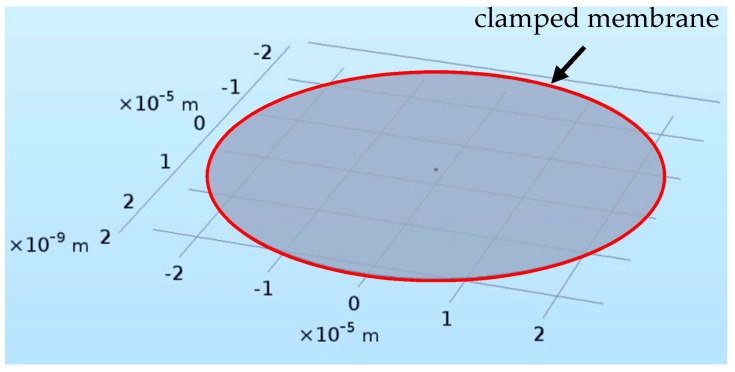
Schematic diagram of the simulated structure.

Regarding the graphene membrane as the resonant element of the F-P probe, the capillary substrate effect of the probe is ignored in the following simulation, which is similar to that in Reference [34]. In this case, the simulated structure is modeled as a clamped circular membrane by COMSOL simulation, as shown in Figure 2. The simulation was performed under vacuum condition. In reality, according to the literatures [35,36,37,38], some researches have widely utilized COMSOL simulation to study the resonant behaviors of monolayer or multilayer graphene diaphragms. Hence, COMSOL Multiphysics 5.3a was introduced in the paper. Firstly, the module “Solid mechanics” was designed to allow thin film to sense the deformation both in the in-plane and out-of-plane directions. Then, the module “Swept mesh” was set to regulate the number, size and distribution of elements in the thickness direction of membrane, thereby ensuring the simulation accuracy. Table 1 lists the simulation parameters of graphene. It is worth mentioning that when a laser hits the surface of graphene membrane, the following three effects will occur: photon pressure, stress change caused by the concentration of photo-generated carrier and opto-thermal effect (thermal stress caused by light). Among them, the last one is the primary factor causing the vibration of graphene membrane. Therefore, another module “Solid heat transfer” was coupled with the module “Solid mechanics” so as to simulate the temperature coupling and the resulting thermal expansion and extraction behaviors. The opto-thermal effect-induced heat in simulation is partially absorbed by graphene membrane, which can be expressed as:

(1) $$Q_{h}=n\cdot \partial \cdot I$$

where *Q_h_* is the absorbed heat; *n* is the layer of graphene membrane; *I* is the intensity of the actuated laser; ∂ = 2.3% is the absorbance of monolayer graphene membrane [25]. It is worth mentioning that for simplification, by assuming uniform membrane thickness, the RF module for confirming the absorption coefficient in COMSOL simulation is not used in this paper. In the future research, the module will be introduced to investigate the influence of the absorption coefficient concerned with non-uniform film thickness on opto-thermal resonance behavior of graphene membrane.

The laser beam generated by the laser S follows Gaussian light distribution so that the intensity (*I*) can be defined as:

(2) $$I=I_{0}\exp(-\frac{r^{2}}{r_{0}^{2}})$$

where *r* is the radius of the membrane; *r*_0_ is the radius of laser beam; *I*_0_ is the peak laser power per unit area at the center of beam spot, which is given by [39]:

(3) $$I_{0}=\frac{P}{\pi r_{0}{}^{2}}$$

where *P* is the output power of laser. In this case, a simulated “heat source” was assigned in the module “Solid heat transfer”, and then, the absorbed heat was determined by Equations (1)–(3). After that, the modules “Steady state study” and “Eigen frequency study” were chosen to achieve the resonance characteristics of graphene membrane imposed on by opto-thermal actuation.

## 3. Simulation on the Effects of Opto-Mechanical Parameters

### 3.1. Structure Properties of Graphene Membrane

**Figure 3:**
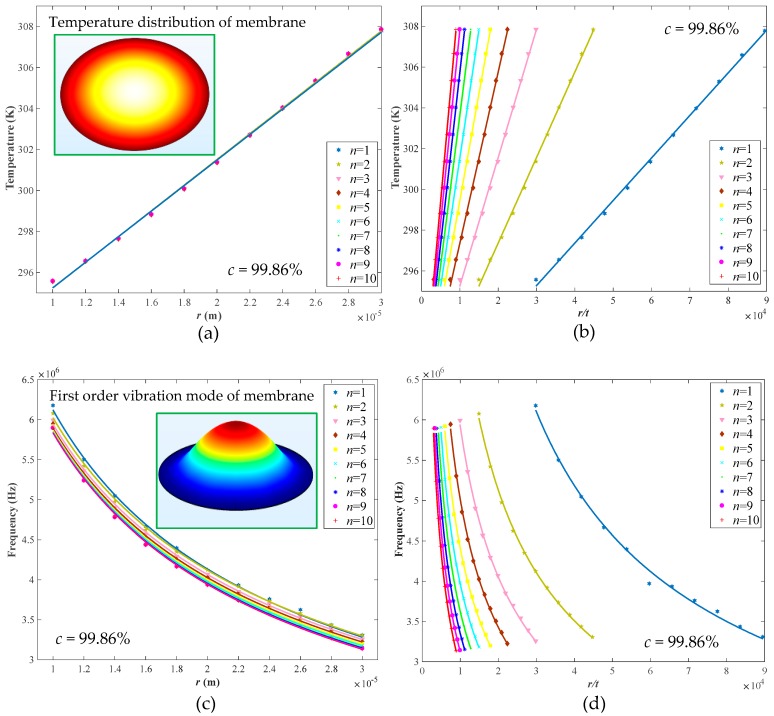
Temperature at the center point of film verse (**a**) radius and layer number of membrane, and (**b**) *r/t*. Natural frequency verse (**c**) radius and layer number of membrane, and (**d**) *r/t*. Note that the symbol *c* represents the correlation coefficient between the simulated data and the corresponding fitted results.

In view of the aspect ratio of graphene, an investigation of the radius and thickness of circular membrane as dominant structural parameters was introduced to evaluate the vibration performance of graphene membrane. We increasingly swept the radius (*r*) in the range of 10~30 μm and thickness (*t*) ranging from 0.335 nm to 3.35 nm (1–10 layers) in COMSOL simulation. As illustrated in the inset of Figure 3a, the temperature caused by opto-thermal effect is uniformly distributed around the center of the membrane such that it decreases from the center point gradually along the radius. Then, the temperature change with the radius (*r*) of graphene membrane was fitted in Figure 3a by a first order polynomial with a correlation coefficient (*c*) of 99.86%, which demonstrates that the temperature in the center point of graphene membrane will increase with radius linearly under the opto-thermal actuation. Additionally, it can be clearly seen from Figure 3a that the temperature has no significant change when the layer of graphene membrane (*n*) varies from monolayer to 10 layers under fixed radii. However, due to the difference in absorption energy and attraction force among different layers, the thickness (or layer number) of graphene has a vital impact on the temperature distribution and resonance frequency of the membrane [40,41,42]. Thus, the radius-to-thickness ratio (*r/t*) was built to further estimate the effect of radius and thickness comprehensively. From Figure 3b, the temperature at the center point of graphene membrane will vary linearly with the ratio (*r/t*); however, the dependence on membrane thickness is dominated. In other words, when the ratio is constant, the temperature will increase sharply with an increase of layer number. This is mainly due to more heats accumulated in a thicker membrane. Furthermore, thicker graphene membrane may strengthen the opacity and absorbance of light on basis of Equation (1) [25,43], such that more energies could be converted from light to heat, which also contributes to the temperature increase.

Referring to the inset of Figure 3c, the natural frequency at first-order vibration mode was confirmed to analyze the effect of radius and thickness of clamped graphene circular membrane on resonance frequency. It can be observed from Figure 3c that the frequency decreases nonlinearly as the radius increases, wherein the frequency-radius curve was fitted by a first order power function with a correlation coefficient of 99.86%. Additionally, a similar variation trend could be found in Figure 3d, wherein, however, an evident distinction was clearly noticed when the layer of membrane varied, especially from monolayer to bilayer. This is primarily because of the interaction between the van der Waals and the vibration direction of each layer [44]. That is to say, the van der Waals interaction between the bilayer and multilayer graphene layers will result in different vibration directions of each layer simultaneously, thereby reducing the vibration frequency. Furthermore, the membrane rigidity tended to increase with the thickness (layer). In this way, graphene membrane with a higher rigidity is expected to resist the deformation via van der Waals coupling between the layers. That also conforms to the fact that monolayer graphene membrane is relatively compliant and apt to bend easily; in contrast, multilayer membrane would be much more rigid. For a certain *r/t*, the thickness of membrane should be a trade-off for achieving high-efficient opto-thermal energy conversion and high natural frequency. Thus, regarding the 50-μm diameter capillary used in the presented F-P resonant probe, the radius of membrane was confirmed as 25 μm to match the capillary, and its thickness was set as 3.35 nm (10 layers) to ensure relative high natural frequency for the subsequent resonant characteristics simulation on F-P resonators with clamped circular graphene membrane.

### 3.2. Film Prestress

**Figure 4:**
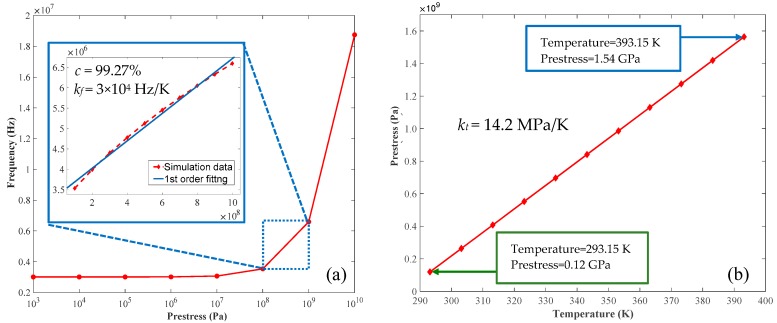
(**a**) Natural frequency verse the prestress of graphene membrane; (**b**) prestress change induced by temperature.

Due to the ultrathin thickness of graphene, the van der Waals force between the film and the substrate will cause strain stretch, which is closely related to the natural frequency-dependent initial prestress of graphene. As a result, the prestress of clamped circular graphene membrane is simulated in the range of 1 × 10^3^ Pa to 1 × 10^10^ Pa, which covers the measured values that may appear in graphene [35]. In accordance with Figure 4a, the obtained resonance frequency has no obvious variation when the film prestress is smaller than 1 × 10^7^ Pa. By contrast, the natural frequency changes from 0.354 × 10^7^ Hz to 1.878 × 10^7^ Hz with a difference of 15.24 MHz when the prestress is larger than 1 × 10^7^ Pa. In consideration of the actual prestress of graphene lower than 10^9^ Pa for this type of resonant probe whose endface is adhered with graphene by van der Waals force [45], the prestress is set in the interval between 10^7^ Pa and 10^9^ Pa. Moreover, in terms of Ref. [46], the natural frequency *f* of circular graphene membrane can be determined as:

(4) $$f=\frac{2.4048}{2\pi r}\sqrt{\frac{n_{0}}{\rho t}}$$

where $n_{0}$ is the initial tension caused by prestress and ρ is the density of graphene. From Equation (4), the natural frequency of circular membrane will increase nonlinearly with the prestress, although it tends to be linear gradually when the prestress is large enough. In order to avoid the nonlinear response of resonant graphene membrane, we further investigated the relation between the natural frequency and the prestress in the local range of 10^8^ Pa to 10^9^ Pa. As depicted in the inset of Figure 4a, the fitted frequency-prestress response with a correlation coefficient of 99.27% represented the natural frequency as an approximated linear function of the prestress. Hence, enhancing the prestress of circular graphene membrane is beneficial to achieve better linearity and the rigidity-dependent detection precision. 

Currently, the typical ways to improve the prestress of graphene relate to reducing the surface roughness of substrate by polishing the endface [12], employing hard-baking technology during fabrication process [47,48,49] and adjusting the environmental temperature. However, it’s difficult for the first two methods to quantitatively control the prestress. As a consequence, with regard to the third method, the correlation between the prestress and environmental temperature was further analyzed by simulation. As shown in Figure 4b, there existed a linear dependence of the initial prestress of graphene membrane on the environmental temperature. When the temperature increased by 100 K from room temperature (293.15 K), the corresponding prestress varied from 1.2 × 10^8^ Pa to 1.54 × 10^9^ Pa (i.e., 0.12–1.54 GPa) with a prestress-to-temperature scale factor (*k_t_*) of 14.2 MPa/K, which turns out that the prestress of graphene membrane can be tuned by adjusting environmental temperatures. Furthermore, in combination with the aforementioned results in Figure 4a, the frequency-temperature scale factor (*k_f_*) was calculated as 3 × 10^4^ Hz/K. Thus, it could be concluded that the natural frequency of graphene membrane enhanced nearly 3 × 10^6^ Hz if the environmental temperature increased to 393.15 K from 293.15 K, which is an extremely remarkable improvement for this type of F-P resonators.

### 3.3. Laser Actuation Parameters

**Figure 5:**
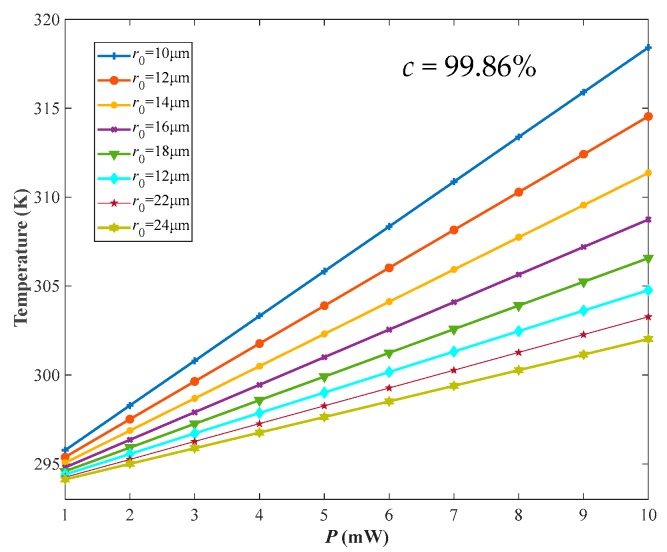
Temperature at the center point of membrane as function of laser output power and light spot radius.

Based on Equations (1)–(3), the light intensity excited by laser is strongly concerned with the laser output power (*P*) and light spot radius (*r*_0_), which reflects the total energy absorbed by graphene membrane. However, it is important to point out that the suspended graphene diaphragm will be partially burn out when the temperature at the center of the membrane is too high. This will definitely disable the F-P resonant probe and then lead to the absence of resonance effect. Generally speaking, the damage threshold of graphene membrane is in connection with the fabrication and transformation process due to the presence of the pretension of transferred graphene membrane. In view of our previous experiments, the output power was kept below 10 mW to avoid the physical damage of graphene membrane mentioned above. Hence, herein we set *P* and *r*_0_ in the range of 1-10 mW and 5–25 μm, respectively. As shown in Figure 5, the temperature at the center point increased linearly with *P* spanning from 1 mW to 10 mW, which had a same variation tendency as the influence of *r/t* on the temperature with a correlation coefficient of 99.86%. Particularly, it should be mentioned that *r*_0_ exhibited a more remarkable effect on the surface temperature of graphene membrane under a large laser output power, and the temperature decreased gradually as the radius of the laser became large. In light of the relation between *r*_0_ and *I*_0_ in Equation (3), the higher the light spot radius is, the weaker the light that approaches the membrane becomes. Furthermore, more surface areas of the suspended membrane will be covered if the light radius gets larger, leading to more temperatures distributed dispersedly and uniformly in the membrane. Therefore, the temperature at the center point may be regulated by controlling the laser output power and light spot radius to generate the enhanced resonance effect while avoiding the damage of suspended graphene diaphragm.

**Figure 6:**
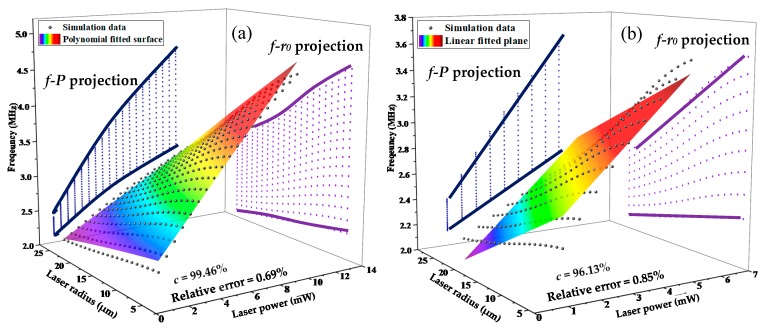
Dependence of resonance frequency on the laser output power and light spot radius by (**a**) polynomial and (**b**) linear fitting methods.

As mentioned above, the change of laser parameters (output power and light spot radius) has an outstanding impact on the resonance frequency induced by temperature. As far as we know, previously reported studies mainly focused on the influences of the laser power on intensity. Nevertheless, the influence of laser output power and laser spot radius on the resonance frequency has not been involved. Thus, by setting the laser output power and light spot radius, respectively, spanning from 1 mW to 10 mW and from 5 μm to 25 μm, the simulated frequency response was achieved as labeled by black sphere in Figure 6a. In order to accurately estimate the dependence of the resonance frequency on laser output power and light spot radius, a second-order polynomial surface was fitted, which was described as:

(5) $$f_{R}=f_{0}+0.2837P-0.0083r_{0}+0.0002r_{0}^{2}+0.0065\cdot P\cdot r_{0}$$

where *f*_R_ is the resonance frequency (MHz); *f*_0_ is the natural frequency (*f*_0_ = 2.3094 MHz). It is generally assumed that the fitted result possesses a good linearity when the relative error is below than 1%. Referring to Figure 6a again, although the correlation coefficient of the fitted surface is up to 99.46% with a relative error of 0.69%, the nonlinear frequency performance will still destroy the accuracy and efficiency of optical detection to a large extent, which is unexpected for resonant sensors. In this case, it is essential to perform a linear approximation for a credible result. From Equation (5), the nonlinear behavior of resonance frequency mainly resulted from the quadratic term of *r*_0_ and the cross term $P\cdot r_{0}$. Furthermore, the curves (*f*-*P* and *f-r*_0_) were also projected in Figure 6a to the corresponding planes according to the simulation data surface, which implied that the frequency as a function of laser output power exhibited a weak nonlinearity. In *f*-*r*_0_ projection especially, the frequency verse laser radius curve showed a bigger nonlinearity than the *f*-*P* curve; however, the nonlinearity reduced gradually with the used smaller laser output power. In this way, the linearity can be promoted by limiting the laser output power range. Figure 6b shows a linear fitted resonance frequency response with a correlation coefficient of 96.13% for the laser output power in a limited range of 1–5 mW. Correspondingly, the resonance frequency as a function of laser output power and light spot radius in this range can be given by:

(6) $$f_{R}=f_{0}+0.2268P-0.0421r_{0}$$

where *f*_0_ = 2.55 MHz. The maximum relative error between the simulated data and the calculated ones by Equation (6) was 0.85%, which is lower than the allowable error of 1% mentioned above. In consequence, Equation (6) is available to approximate the initial output power and radius of laser beam for this type of F-P resonators at room temperature to make the detected signals display a preferable linearity. 

### 3.4. Clamped Boundary Conditions

**Figure 7:**
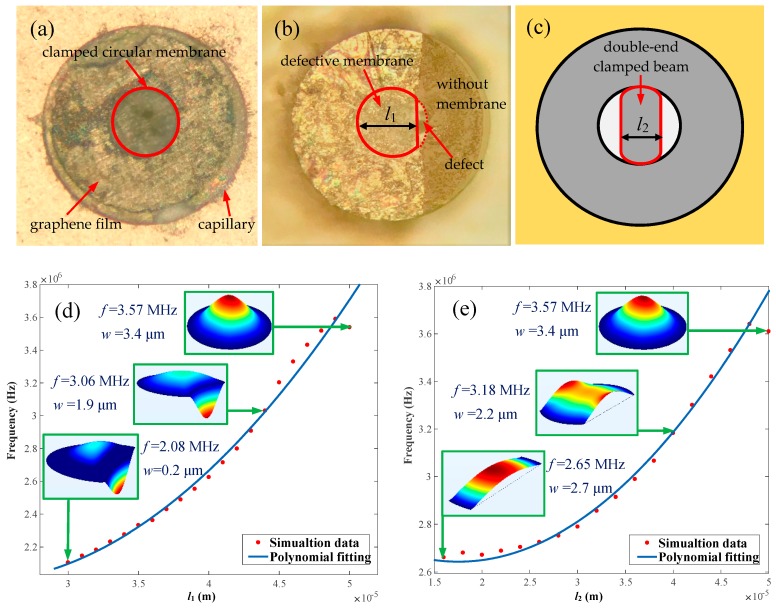
Boundary condition analysis of an F-P resonant probe with circular suspended graphene membrane. Pictures of (**a**) circular graphene membrane and (**b**) unilateral edge defective membrane, and (**c**) schematic diagram of probe with a bilateral symmetry edge defect. Frequency variation versus the residual width of defective membrane corresponding to (**d**) *l*_1_ and (**e**) *l*_2_, respectively.

When an intact graphene film (Figure 7a) is successfully transferred to the resonant F-P probe, it is well clamped on the capillary (ferrule), which enables a comparatively excellent vibration mode. This type of film boundary condition is beneficial to detecting the deflection (*w*) at the center point of film by the laser. However, during the process of transferring graphene to capillary endface, due to the imperfect attaching and clamping of circular membrane, certain boundary defects will appear as displayed in Figure 7b. Obviously, the resonance performance of resonator under such a condition will be deteriorated. For the sake of evaluating the availability of the circular graphene membrane even under the condition with boundary defect, we compared the effect of a defective membrane (Figure 7b) on natural frequency with that of a defect-free membrane (Figure 7a). As shown in Figure 7b, since the light spot radius of the laser R in Figure 1a is 5 μm, in combination with the capillary inner diameter of 50 μm, the length (*l*_1_) of the unilateral defective membrane should be at least equivalent to 30 μm so as to ensure the integrity of detected signal irradiated on the membrane central area. As such, when *l*_1_ decreases from 50 μm to 30 μm, meaning a gradual increasing boundary defect as presented in Figure 7d, the natural frequency will decrease by ~1.49 MHz and the position where the maximum vibration mode occurs also deviates from the center of circular membrane, thus resulting in the inferior deflection reduced dramatically. This resulting undesirable phenomenon likely results from the asymmetrical clamped conditions of circular membrane caused by individual defects. In view of this problem, we set a symmetrical clamped condition formed by a bilateral symmetry boundary defect as shown in Figure 7c. Then, Figure 7e illustrates the decreasing natural frequency of membrane as a function of the bilateral defect length (*l*_2_), as well as the deflection of membrane. However, the first order vibration mode occurs at the central area of film instead of the edge of membrane until the length to width ratio is larger than 5. The phenomenon can be explained by the fact that the circular membrane becomes a clamped beam, which enables a higher-amplitude deflection than previous defective circular membrane, corresponding to an outstanding *Q* factor despite of lower natural frequency. That is to say, the circular graphene membrane can be fabricated to a graphene beam by photoetching or ion etching to achieve a remarkable detected signal and relatively high *Q* factor for F-P resonators. To sum up, the utilization and reliability of the resonant probe with a graphene membrane can be improved to a great extent, despite the lower successful rate of well transferring and attaching graphene film on a capillary endface with a hole no less than 50 μm in diameter.

## 4. Conclusions

A photo-thermally excited Fabry-Perot resonant probe with suspended clamped circular graphene membrane of 50 μm in diameter was proposed, and its opto-mechanical vibration behaviors were investigated by COMSOL simulation in this paper. The influence of the radius-to-thickness ratio of film on the optical fiber light-induced temperature absorbed by suspended graphene membrane exhibited an excellent fitted linearity with a correlation coefficient of 99.86%. Moreover, in order to achieve an enhanced linear vibration response, the film prestress could be adjusted in the range of 0.1–1 GPa by tuning environmental temperature based on the interaction between film thermal strain and substrate adhesion. In contrast to previously reported studies that primarily focused on the effect of laser output power on light intensity, this paper evaluated the dependence of the laser parameters (output power and light spot radius) on the temperature distribution and natural frequency of graphene membrane. More importantly, adverse clamped boundary conditions, such as edge defect and asymmetry clamped prestressed membrane, were improved by fabricating unstable defective circular membrane to stable double-ended clamped beam. Therefore, further research on the preferable trade-off between the opto-mechanical characteristic parameters and boundary conditions of suspended film acting as a reflective surface is needed to optimize opto-thermally actuated graphene resonators with an F-P micro cavity.

## Figures and Tables

**Table 1:** Initial simulation parameters for circular graphene membrane.

Description	Value	Unit
Diameter	50	μm
Thickness	3.35	nm
Density [10]	2208	kg/m^3^
Young modulus [10]	1.1	TPa
Poisson ratio [13]	0.41	
Thermal conductivity [29]	5300	W/(m·K)
